# Virgin Coconut Oil Supplementation Prevents Bone Loss in Osteoporosis Rat Model

**DOI:** 10.1155/2012/237236

**Published:** 2012-09-16

**Authors:** Zil Hayatullina, Norliza Muhammad, Norazlina Mohamed, Ima-Nirwana Soelaiman

**Affiliations:** Pharmacology Department, Faculty of Medicine, Universiti Kebangsaan Malaysia, 50300 Kuala Lumpur, Malaysia

## Abstract

Oxidative stress and free radicals have been implicated in the pathogenesis of osteoporosis. Therefore, antioxidant compounds have the potential to be used in the prevention and treatment of the disease. In this study, we investigated the effects of virgin coconut oil (VCO) on bone microarchitecture in a postmenopausal osteoporosis rat model. VCO is a different form of coconut oil as it is rich with antioxidants. Three-month-old female rats were randomly grouped into baseline, sham-operated, ovariectomized control (Ovx), and ovariectomized rats fed with 8% VCO in their diet for six weeks (Ovx+VCO). Bone histomorphometry of the right femora was carried out at the end of the study. Rats supplemented with VCO had a significantly greater bone volume and trabecular number while trabecular separation was lower than the Ovx group. In conclusion, VCO was effective in maintaining bone structure and preventing bone loss in estrogen-deficient rat model.

## 1. Introduction

Osteoporosis is a metabolic disorder which is characterized by deterioration of bone tissue and loss of bone mass with a consequent increased risk of fracture. It is due to decreased activities of osteoblasts and increased activities of osteoclasts [1]. In both sexes, estrogens and testosterones play a vital role in the pathophysiology of osteoporosis. Postmenopausal osteoporosis which occurs in aging women is usually associated with estrogen deficiency [2]. The absence of ovarian hormone will result in accelerated bone resorption by osteoclasts and reduced bone formation by osteoblasts [3]. Oxidative stress has been implicated in the pathogenesis of osteoporosis as evidenced by numerous *in vitro* and *in vivo* studies [4, 5]. Oxidative stress occurs when the body's antioxidant defense is unable to overcome the cellular damage caused by free radical molecules [6]. Supplementation with antioxidants like vitamins C [7] and E [8, 9] has been shown to prevent bone loss in osteoporosis. On the other hand, deficiency in vitamins E and D may cause a decrease in cartilage cells and osteocytes, as well as thinning of the cortex and trabecular in mice [10].

Virgin coconut oil (VCO) is obtained from fresh, mature coconut kernel without the use of heat and without undergoing refining process [11]. This retains the important biologically active components in the oil such as antioxidant vitamins and phenolic compounds. VCO supplementation in diet has been shown to reduce the cholesterol and triglyceride levels, maintain blood coagulation factors, and prevent oxidation of low-density lipoprotein lipids [12, 13]. Besides, VCO has been reported to have anticancer, antimicrobial, and anti-inflammatory properties [14–16]. Another study showed that virgin coconut oil lowered alcohol-induced oxidative stress by reducing testicular malondialdehyde level (tMDA) and ameliorated the deleterious effect of alcohol on serum testosterone level in rats [17]. Diet supplemented with virgin coconut oil was shown to increase the antioxidant status in rats [18]. To date, there has been no research done on the effects of VCO on bone metabolism.

Rats are widely accepted animal model for studying bone diseases as the bone remodelling and resorption processes in young and adult rats resemble those of human [19]. The responses of bone towards mechanical stress, hormones, and drugs are similar in rat and human [20]. Therefore, ovariectomized rat is a useful model for osteoporosis since the progressive loss of bone matrix is similar to that in postmenopausal women with osteoporosis [21]. In the present study, we investigated the effects of virgin coconut oil supplementation on bone microarchitecture of ovariectomized rats. We carried out bone histomorphometry analysis on undecalcified bone sections in order to obtain information about the trabecular structure. 

## 2. Materials and Methods 

### 2.1. Preparation of Virgin Coconut Oil (VCO)

The preparation of virgin coconut oil was modified from previous studies by Nevin and Rajamohan 2004, 2006 [12, 18]. To summarize the procedure, mature coconuts were bought from a local market and the fresh coconut meat was grated using an electric grater. A 100 g of grated coconut flesh was mixed with 200 g of natural coconut water. The coconut mixture was squeezed into viscous slurry until all the creamy milk was obtained. The coconut milk was left in room temperature for 48 hours to allow fermentation process to take place. At the end of the second day, there would be three layers seen with the virgin coconut oil forming the second layer. The oil was then gently scooped out and filtered to remove the coconut residue. Lastly, the oil was separated from the bottommost aqueous layer using a separatory funnel.

### 2.2. Experimental Design

A total of 40 female Wistar rats aged 3 months old, weighing between 200–250 g, were obtained from Universiti Kebangsaan Malaysia Animal Centre. The animals were allowed a two-week acclimatization period during which they were fed on commercial rat chow (Gold Coin, Klang Selangor, Malaysia) and deionized water given *ad libitum*. Rats were accommodated two per cage at room temperature with a 12 hours light/dark cycle. The rats were randomly divided into five groups with eight rats in each group: Baseline, Sham, ovariectomized control (Ovx), ovariectomized and treated with virgin coconut oil (Ovx+VCO), and ovariectomized and given calcium (Ovx+Ca). Rats in the baseline group were euthanised at the start of the experiment. The ovariectomized rats had their ovaries removed under anaesthesia. The abdomen of the sham rats were operated on and their ovaries were manipulated but were left intact. Treatment commenced two weeks after ovariectomy to allow ample time for healing. The Ovx+Ca rats received 1% calcium in their drinking water. All the rats except the Ovx+VCO group were fed with standard rat chow diet. The diet for Ovx+VCO rats contained 8% virgin coconut oil mixed with the standard rat chow [18]. The diet was prepared by mixing 8 g VCO into 100 g standard rat chow. Gain in body weight was recorded weekly. Treatment lasted for six weeks after which the rats were euthanised by overdoses of Phenobarbital (400 mg/kg) and their right femora removed for bone histomorphometry analysis. This study was approved by the Animal Ethics Committee of Universiti Kebangsaan Malaysia (UKMAEC: PP/FAR/2009/NORLIZA/24-FEBRUARY/250-MARCH-2009-JULY-2010).

### 2.3. Bone Histomorphometry

 At sacrifice, the right femora were fixed in 10% formalin. After fixation, the bones were cut at the midshaft using a rotary electronic saw (Black & Decker, USA). The distal femora were then halved longitudinally and subsequently dehydrated in graded concentrations of ethanol. They were then further processed for embedding in methyl methacrylate polymer according to the manufacturer's instructions (Osteo-Bed Bone Embedding Kit; Polysciences, USA). Following that, the femora were sectioned using a Manual Rotary Microtome (Model 2235, Leica, Germany) and serial bone sections at 7 microns thick were obtained. The undecalcified bones were stained using von Kossa method [22]. Histomorphometric measurements were carried out on the secondary spongiosa of the distal femoral metaphysis at a distance between 3 mm and 7 mm from the lowest point of growth plate and from 1 mm of the bilateral cortices. Measurements were made at 4x objective magnification using a light microscope (Leica, Germany) connected to an image analyzer (Image Pro-Express, Media Cybernatics, USA). The parameters measured in this study were trabecular bone volume, trabecular thickness, trabecular number, and trabecular separation. Trabecular bone volume (BV/TV) is the amount of trabecular bone within the spongy space (expressed as a percentage). BV/TV is derived from measurements of bone area (B.Ar) and cancellous tissue area (T.Ar) and calculated as BV/TV = 100 × B.Ar/T.Ar. Trabecular thickness (Tb.Th, in micrometers) was derived from trabecular perimeter (B.Pm) and B.Ar (Tb.Th = 1.99 × B.Ar/2/B.Pm). Trabecular number (Tb.N, expressed per millimeter) and trabecular separation (Tb.Sp, expressed per micrometer) were calculated assuming that trabecular bone can be modeled by the parallel plates and bar model (Tb.N = Tb.Ar × 10/Tb.Th; Tb.Sp = 1000/Tb.N − Tb.Th). All the formula, symbols, units, and nomenclature used for bone histomorphometry in this study were in accordance to the recommendation by the American Society for Bone and Mineral Research (ASBMR) Histomorphometry Nomenclature Committee [23].

### 2.4. Statistical Analysis

Statistical analysis was performed using Statistical Package for Social Science software (SPSS version 19.0; SPSS). Results were expressed as mean ± standard error of the mean (SEM) with significance defined as *P* value of 0.05 or less. Data were tested for normality using the Kolmogorov-Smirnov test. For normally distributed data, analysis of variance (ANOVA) followed by Tukey's HSD test was carried out. Kruskal-Wallis and Mann-Whitney tests were used for data that were not normally distributed.

## 3. Results

### 3.1. Body Weight

**Figure 1:**
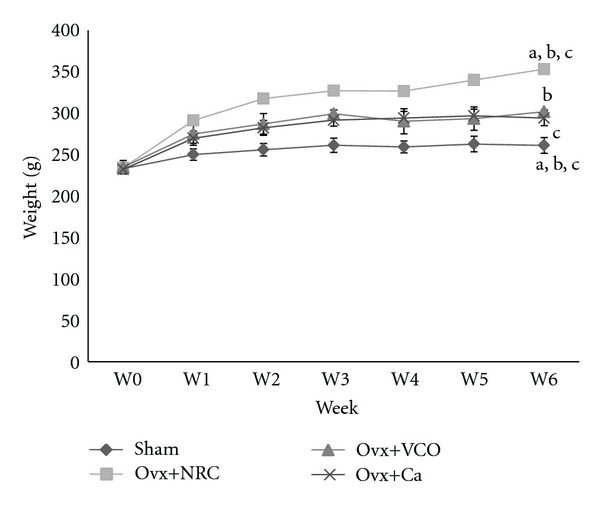
Weight of the rats throughout six-week study period. Same letters indicate significant difference between groups (*P* < 0.05). Data are expressed as mean ± SEM. Sham: sham-operated; Ovx: ovariectomized control rats; Ovx+VCO: ovariectomized rats with virgin coconut oil; Ovx+Ca: ovariectomized rats with calcium.

The body weight gain pattern is shown in Figure 1. By the end of the sixth week, the ovariectomized control rats gained significant weight compared to all other groups. The ovariectomized rats treated with virgin coconut oil and calcium had significantly higher body weight than Sham rats (Figure 1).

### 3.2. Trabecular Bone Volume (TV/BV)

**Figure 2:**
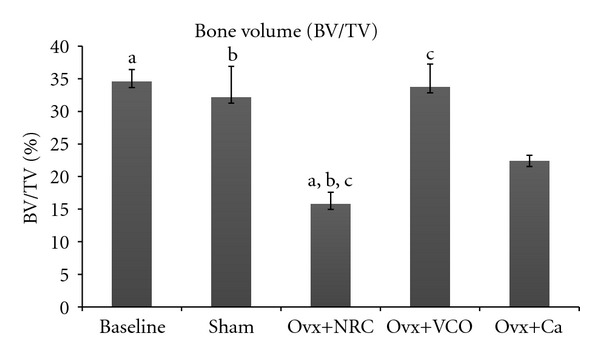
Bone volume (BV/TV). Same letters indicate significant difference between groups (*P* < 0.05). Data are expressed as mean ± SEM. Sham: sham-operated rats; Ovx: ovariectomized control rats; Ovx+VCO: ovariectomized rats with virgin coconut oil; Ovx+Ca: ovariectomized rats with calcium.

Ovariectomized control rats had a reduced trabecular bone volume compared to the Baseline, Sham, and Ovx+VCO groups. The Ovx+VCO group had a significantly higher TV/BV than the Ovx group whilst remained comparable with the Sham group (Figure 2). However, there was no significant difference between Ovx group and Ovx+Ca rats. Treatment with calcium did not have any effect on trabecular volume (Figure 2).

### 3.3. Trabecular Number (Tb.N)

**Figure 3:**
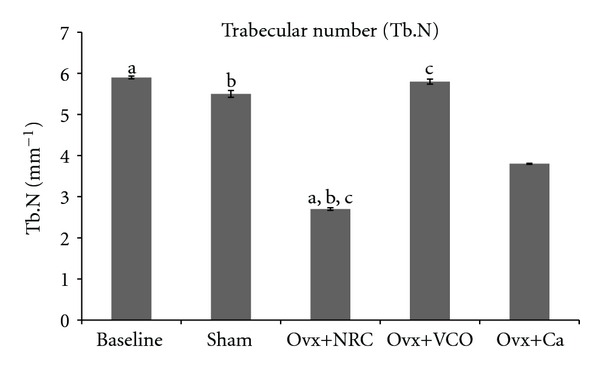
Trabecular number (Tb.N). Same letters indicate significant difference between groups (*P* < 0.05). Data are expressed as mean ± SEM. Sham: sham-operated; Ovx: ovariectomized control rats; Ovx+VCO: ovariectomized rats with virgin coconut oil; Ovx+Ca: ovariectomized rats with calcium.

The Ovx group had reduced Tb.N compared to Baseline, Sham and Ovx+VCO groups. Treatment with VCO significantly prevented the reduction in Tb.N. There was no significant difference in Tb.N between the Ovx+VCO, Baseline and Sham groups. However, treatment of Ovx rats with calcium failed to prevent the reduction in Tb.N as there was no significant difference in Tb.N between the Ovx and Ovx+Ca groups (Figure 3). 

### 3.4. Trabecular Thickness (Tb.Th)

**Figure 4:**
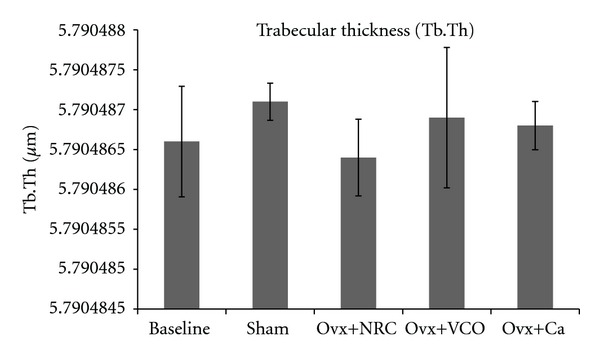
Trabecular thickness (Tb.Th). Same letters indicate significant difference between groups (*P* < 0.05). Data are expressed as mean ± SEM. Sham: sham-operated; Ovx: ovariectomized control rats; Ovx+VCO: ovariectomized rats with virgin coconut oil; Ovx+Ca: ovariectomized rats with calcium.

Ovariectomy did not reduce the value of Tb.Th. There was no significant difference noted between Ovx and the other four groups of rats. There was no difference either among the Ovx+VCO, Ovx+Ca, Baseline, and Sham groups (Figure 4). 

### 3.5. Trabecular Separation (Tb.Sp)

**Figure 5:**
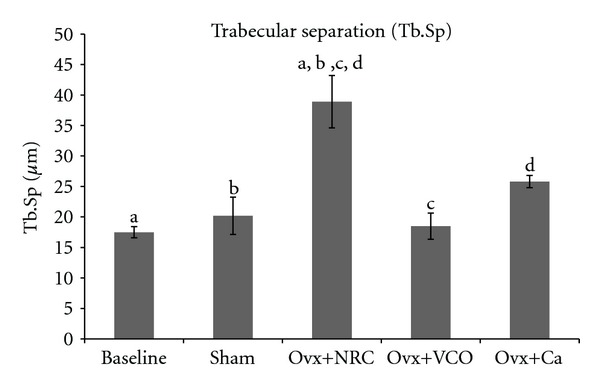
Trabecular separation (Tb. Sp). Same letters indicate significant difference between groups (*P* < 0.05). Data are expressed as mean ± SEM. Sham: sham-operated; Ovx+NRC: ovariectomized control rats; Ovx+VCO: ovariectomized rats with virgin coconut oil; Ovx+Ca: ovariectomized rats with calcium.

Rats in the Baseline, Sham, Ovx+VCO, and Ovx+Ca groups had significantly lower Tb.S compared to Ovx group. There was no significant difference between the Baseline, Sham, Ovx+VCO, and Ovx+Ca rats (Figure 5).

### 3.6. Bone Histology

The trabecular bones of the Ovx group appeared to be sparser compared to the other groups. The bones of Ovx+VCO group appeared thicker and denser than the Ovx group and comparable to the Sham.

## 4. Discussion

Loss of estrogen contributes to increased body weight in ovariectomized rats (Figure 1). Estrogen deficiency induces hyperphagia in rats [24] as lack of estrogen leads to reduce leptin secretion from the adipose tissues. Leptin, a 14 kDa protein, acts on the hypothalamus to regulate food intake and a low level of it will send signals to the body to increase intake of food [25]. Treatment with VCO and calcium also contributes to weight gain when compared to the sham operated rats. However, the rats in these two groups did not gain as much weight as rat in the Ovx group. Studies involving male rats fed with VCO did not show any significant weight gain compared to control rats [12, 13]. To date, there have not been any studies investigating the effect of VCO on change of weight in estrogen-deficient animals. The effects of calcium on body weight have been so far inconsistent. Our previous study [26] showed that ovariectomized rats receiving calcium in drinking water had a much higher weight gain similar to the Ovx group. The slight increase in rats' body weight in the VCO and calcium groups in the present study could still be due to increased food intake as a result of lower leptin secretion though the impact is less severe compared to Ovx rats. 

In the present study we evaluated the effects of six-week virgin coconut oil (VCO) supplementation on bone microarchitecture in ovariectomized rats based on bone histomorphometry. Histomorphometry is an important technique for evaluating the rate of bone turnover and for examining bone quality and architecture [27]. Bone histomorphometry is also used diagnostically in metabolic bone diseases such as hyperparathyroidism, Paget's disease, and osteoporosis [28]. The six-week study period was chosen based on our previous studies which showed that the changes in bone turnover markers were detected as early as the fourth week after exposure to oxidative stress [9] and that histomorphometric changes in ovariectomized rats were already evidenced six weeks postovariectomy [29].

**Figure 6:**
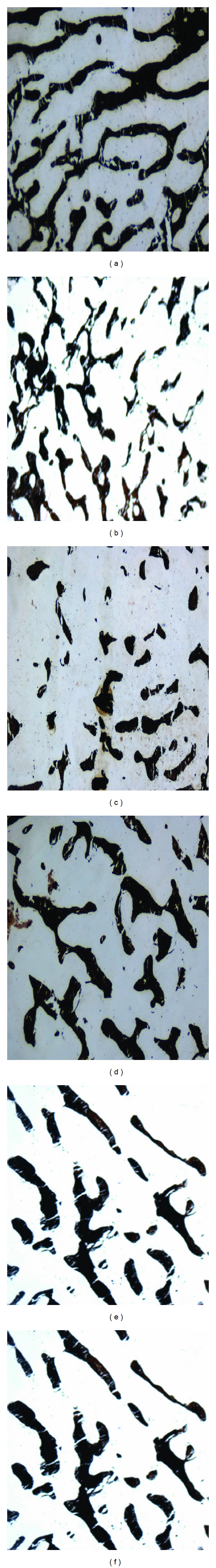
Photomicrographs of undecalcified bone sections stained with von Kossa. (a) Baseline; (b) Sham; (c) ovariectomized control rats (Ovx); (d) ovariectomized rats with virgin coconut oil (Ovx+VCO); (e) ovariectomized rats with calcium (Ovx+Ca). (20X magnification.) The trabecular bones of Ovx+NRC appeared to be sparser compared to the other groups. The bones of Ovx+VCO group appeared thicker and denser than Ovx group and comparable to Sham group.

The results of our study revealed that the bone volume and trabecular number in ovariectomized control rats were significantly lower than those in the Baseline, Sham and Ovx+VCO groups. Ovariectomized rats also had higher trabecular separation compared to the other rats (Figures 2, 3, and 5). Consistent with the previous report on changes in lumbar vertebral bodies of ovariectomized rats [30], the trabecular bones in Ovx group were less in number and arranged in a more spaced out fashion with clear evidence of bone loss (Figure 6(c)). In addition, our study did not show any changes in the value of trabecular thickness in Ovx group (Figure 4). This meant that the loss of bone was mainly due to trabecular perforation and loss of trabecular connectivity; not due to trabecular thinning. Trabecular perforation may therefore pose an increased bone fragility [31] associated with an increase in systemic bone turnover markers at the tissue level [30]. Our observation is slightly different from that of Bagi et al. [32] whereby in aged ovariectomized rats, the bone loss was associated with trabecular thinning, reduction in trabecular number and increase in trabecular separation. Our results however were consistent with previous studies performed in mature ovariectomized rat model [33, 34] whereby trabecular perforation is the main mechanism of bone loss at the early stage of oestrogen deficiency [35]. 

 Estrogen is essential for normal skeletal development and maintenance of bone health in both men and women. Estrogen deficiency causes osteoblasts to release interleukin-1 (IL-1) and interleukin-6 (IL-6) which stimulate the differentiation and activation of osteoclasts, leading to increased bone resorption [36–38]. Other than exerting its endocrine function, estrogen has been found to have antioxidant property [39, 40]. As a consequence, lack of estrogen will increase lipid peroxidation and free radical formation [41], which will hinder the functions of bone cells particularly the osteoblasts and the osteoclasts. Oxidative stress damages the osteoblasts and accelerates bone loss by increasing osteoclastogenesis [42]. Bone loss and osteoporosis can be prevented if oxidative stress is minimized. Antioxidants such as vitamin E can protect bone cells from the damaging effect of oxidative stress as shown by *in vivo* studies using estrogen-deficient laboratory animals [8, 41]. Another study using vitamin C revealed that daily intake of this antioxidant vitamin increased the bone mineral density (BMD) of postmenopausal women [43].

Treatment of ovariectomized rats with VCO seems to reverse the effects of estrogen deficiency on the bone structure. The bone volume and trabecular number were significantly higher than the ovariectomized control group (Figures 2 and 3). The six-week treatment of VCO in ovariectomized rats completely restored trabecular bone volume back to the Sham group level. The trabecular bones of the VCO group were more closely connected to each other as the trabecular separation value was lower compared to the ovariectomized control rats. Photomicrograph of the trabecular bone in VCO-treated rats appeared similar to the Sham rats (Figure 5). This indicates that the administration of virgin coconut oil prevented bone loss and maintained bone microarchitecture of estrogen-depleted rats. Virgin coconut oil completely prevented the bone loss by preserving trabecular bone mass and trabecular network connectivity in the metaphyseal region. 

The effects of VCO on bone microarchitecture was much better than treatment with calcium as the latter only prevented the reduction in trabecular separation (Figure 5) but failed to increase the bone volume and trabecular number (Figures 2 and 3). The superior effect of VCO over calcium was probably because VCO contains high amount of saturated fats in the form of medium chain triglycerides (MCTs) [44]. Saturated fats are important for calcium absorption from the intestines [45]. Our findings supported the recommendation that calcium therapy alone is not sufficient for the treatment of osteoporosis [46]. Other researchers also observed that calcium supplementation given to ovariectomized rats still resulted in lower bone volume and trabecular number as compared to sham-operated rats [47]. In yet another study using ovariectomized rats, calcium supplementation was shown to improve fracture healing but failed to improve bone strength [48]. 

VCO and coconut oil obtained from copra (dried kernel meat of coconut) contain the same composition of triglycerides with more than 90% being short- and medium-chain saturated fatty acids while the remaining are the unsaturated fats [44, 49]. However, VCO is different from coconut oil as the former contains a lot more biologically active components like vitamins and polyphenols [12]. Marina et al. showed that VCO has higher antioxidant capacity than copra oil and alpha-tocopherol [50]. This corresponds to the higher amount of polyphenols in VCO. Phenolic compounds are found to be associated with many health benefits [51, 52]. They exhibit a wide range of physiological properties such as antiallergenic, antiaftherogenic, anti-inflammatory, antimicrobial, antithrombotic, cardioprotective, and vasodilatory effects [53–57]. 

To the best of our knowledge, we are the first to report the ability of VCO in preventing osteopenia in postmenopausal osteoporosis rat model. The beneficial effects of virgin coconut oil on bone microarchitecture may be due to its antioxidant property. We propose that the antioxidant components in VCO prevented the free-radical-induced bone loss associated with estrogen deficiency. The preventive effects of VCO could be explained by the various bioactivities of the many phytochemicals found in the oil, particularly the polyphenols. Further studies are required to determine the exact mechanism on how VCO and polyphenols provide beneficial effects on bone health. It could be due to its direct antioxidant activity or combination with other mechanisms involving osteoclastogenesis and modulation of osteoblast function. Transcription factors such as NF-*κ*B and activator protein-1 (AP-1) as well as cellular signalling pathways such as mitogen-activated protein kinase (MAPK), bone morphogenetic protein (BMP), oestrogen receptor and osteoprotegerin/receptor activator of NF-*κ*B ligand (OPG/RANKL) may be implicated [58]. The effects of VCO on bone formation rate, other dynamic and cellular parameters as well as on bone turnover markers, would be helpful to provide insights to the mechanisms involved. Information on how VCO affect bone strength, bone density and mineral composition should also be sought after. 

The effects of VCO in preventing bone loss were similar to those seen in other experimental osteoporosis studies using other plants which are rich in polyphenols. Phloridzin, an apple polyphenol, green tea polyphenol, and oleuropein, a polyphenol found in olive oil, are few examples of plant polyphenols which exert bone sparing activity ranging from increasing bone mineral density, increasing osteoblast function, improving bone mechanical strength to reducing bone resorption markers [59–61].

Due to the many benefits of VCO, researchers are yet to determine accurately how much coconut oil is needed daily to gain optimal health. The dose that we chose to use in this study is equivalent to the human dose (3.5 tablespoons daily of VCO for a 72 kg man) as recommended by Isaacs and Thormar [62]. This was derived from the amount of medium chain fatty acid (MCFAs) present in human breast milk which is the perfect formula that gives infants protective shield from infections and other illnesses. The 60% composition of MCFAs in virgin coconut oil is almost similar to that in human breast milk [62]. 

To summarise our findings, virgin coconut oil effectively improved bone structure and prevented bone loss in osteoporosis animal model. The beneficial effects of VCO on bone microarchitecture may be due to the high polyphenols which exert antioxidant property. Virgin coconut oil could offer an interesting approach to prevent accelerated bone loss in osteoporosis especially in postmenopausal women.
